# Characterization of the complete plastid genome of *Butia eriospatha* (Arecaceae)

**DOI:** 10.1590/1678-4685-GMB-2020-0023

**Published:** 2020-09-11

**Authors:** Jeison Willy de Souza Magnabosco, Hugo Pacheco de Freitas Fraga, Raquel Santos da Silva, Marcelo Rogalski, Emanuel Maltempi de Souza, Miguel Pedro Guerra, Leila do Nascimento Vieira

**Affiliations:** 1 Universidade Federal do Paraná, Programa de Pós-graduação em Botânica, Curitiba, Paraná, Brazil.; 2 Universidade Federal de Viçosa, Programa de Pós-graduação em Fisiologia Vegetal, Viçosa, MG, Brazil.; 3 Universidade Federal do Paraná, Programa de Pós-graduação em Bioquímica e Biologia Molecular, Curitiba, PR, Brazil.; 4 Universidade Federal de Santa Catarina, Programa de Pós-graduação em Recursos Genéticos Vegetais, Florianópolis, SC, Brazil.; 5 Universidade Federal de Santa Catarina, Programa de Pós-Graduação em Ecossistemas Agrícolas e Naturais, Curitibanos, SC, Brazil.

**Keywords:** Palm, atlantic rainforest, plastome, molecular evolution

## Abstract

*Butia eriospatha* is an endemic palm species from the Atlantic Rainforest in Brazil, a biodiversity hotspot. This species is currently listed in the IUCN red list as vulnerable and lacks specific plastid markers for population genetics studies. In addition, the evolutionary relationship within the genus *Butia* is not yet well resolved. Here, we sequenced and characterized the complete plastid genome (plastome) sequence of *B. eriospatha*. The complete plastome sequence is 154,048 bp in length, with the typical quadripartite structure. This plastome length and genes content is consistent with other six species from tribe Cocoseae. However, the Inverted Repeat (IR) borders show some variation among the analyzed species from this tribe. Species from the Bactridinae (*Astrocaryum* and *Acrocomia*) and Elaeidinae (*Elaeis*) subtribes present the *rps19* gene completely duplicated in the IR region. In contrast, all plastomes sequenced from the subtribe Attaleinae (*Butia*, *Cocos*, *Syagrus*) present one complete CDS of *rps19* and one partial copy of *rps19.* The difference in the IR/LSC junctions between Attaleinae and the sister clades Bactridinae + Elaeidinae might be considered an evolutionary signal and the plastome sequence of *B. eriopatha* may be used in future studies of population genetics and phylogeny.

The family Arecaceae, comprises approximately 188 genera and 2,585 species, distributed throughout tropical and subtropical climate of the world, but it is most diverse in tropical forest habitats, being present from Americas to Asia-Pacific region (Palmweb).

*Butia* (Becc.) Becc. is a genus of the family Arecaceae, indigenous from South America. This genus includes 18 species (WFO, 2019), among them *Butia eriospatha* (Mart. ex Drude) Becc.. This species is endemic to southern Brazil and occurs in the Atlantic Rainforest phytogeographic domain: mainly in high altitude grassland, grassland, palm grove (Heiden *et al.*, 2020). *Butia eriospatha* is easily distinguished by its solitary stem with 4-5 meters in hight, globose fruit, spathe ferruginous-pubescent on outer surface, and pistillate flowers with 3-7 mm (Deble *et al.*, 2011; Heiden *et al.*, 2020). Among palms, *B. eriospatha* has a high economic importance, being used for ornamentals projects, and with edible fruits. Currently, the fruits are consumed fresh or in pulp, alcoholic drinks, jams, jellies or ice cream (Hoffmann *et al.*, 2014).

Species of the genus *Butia* show the same chromosome number (2n = 2x = 32) (Correa *et al.*, 2019) and the genus is well supported as monophyletic (Meerow *et al.*, 2015). However, within *Butia,* few molecular markers were used in phylogenetic studies. The most recent study on *Butia* phylogeny used only one plastid marker (*trnH-psbA*) and two nuclear markers (ITS and WRKY19) and failed to resolve the evolutionary relationship between *Butia* species (Pereira, 2019).

Plastome sequences are frequently used to understand evolutionary events and efficiently resolve phylogenetic relationships (Lopes *et al.*, 2018, 2019). Typically, the plastome is represented as a single circular molecule composed by two inverted regions (IR), a large single copy (LSC) and a small single copy (SSC), with 120 to 130 genes, encoding ribosomal RNA (rRNA), transfer RNA (tRNA) and peptides. The plastome size varies from 107 kb to 218 kb, depending on each species (Daniell *et al.*, 2016; Menez*e*s *et al.*, 2018).

Here we report the complete plastome of *B. eriospatha* and describe its structure and gene composition. For this, *B. eriospatha* leaves were collected from a single individual at the Federal University of Santa Catarina - Brazil. DNA isolation with plastid DNA-enrichment was performed according to Vieira *et al.* (2014). The DNA was quantified using Qubit^®^ fluorometer (Invitrogen, Carlsbad, CA) and 1 ng of DNA was used for preparing sequencing libraries with Nextera XT DNA Sample Prep Kit (Illumina Inc., San Diego, CA). Libraries were sequenced on Illumina MiSeq (Illumina Inc.). The obtained paired-end reads (2 x 300 bp) were used for *de novo* assembly in CLC Genomics Workbench 8.0v. The plastome annotation was performed using DOGMA software (Wyman *et al.*, 2004) followed by manual curation using Geneious (Kearse *et al.*, 2012). The graphical map of plastomes was generated by OrganellarGenomeDRAW (OGDRAW) (Greiner *et al.*, 2019). The complete nucleotide sequence of *B. eriospatha* (MN329806) plastome was deposited in the GenBank database. The junction site of plastomes (LSC/IR, IR/SSC) from the tribe Cocoseae (Arecaceae: Arecoideae) were visualized and compared using IRScope (Amiryousefi *et al.*, 2018).

*Butia eriospatha* plastome is a circular molecule with 154,048 bp in length and the typical quadripartite structure, including a large single copy with 83,805 bp, two inverted repeat regions (26,437 bp) and a small single copy with 17,369 bp (Figure 1). This plastome assembly was realized with 457,423 plastid reads (all trimmed with 0.05 quality scores in CLC Genomics Workbench), which resulted in approximately 820x genome coverage depth. The minimum genome coverage was 206x, which shows the high coverage sequencing of this plastome.

**Figure 1:**
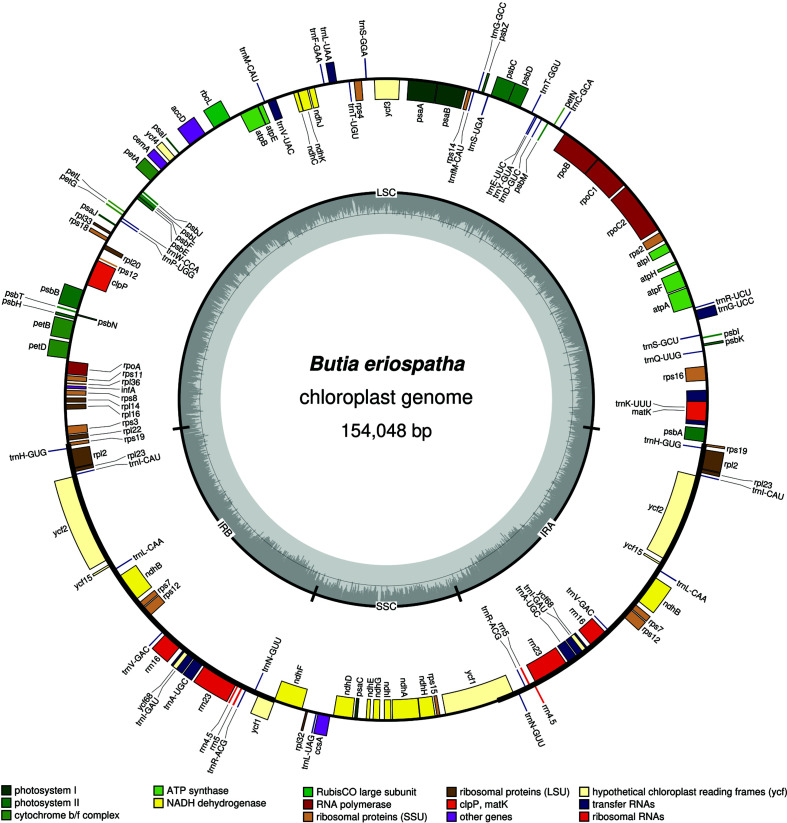
Graphical map of *Butia eriospatha* plastid genome. Genes shown on the outside of the circle are transcribed clockwise; genes on the inside are transcribed counterclockwise. Genes were grouped and represented by different colors according their function group. The GC content is represented by the dashed darker grey area in the inner circle, the lighter grey area represents AT content.

This plastome encodes 113 unique genes, being 79 protein-code genes, 30 tRNA genes and four rRNA genes (Table 1), the same number as the other Cocoseae species (Lopes *et al.*, 2018, 2019). The 20 duplicated genes in the IRs are: eight protein-coding genes (among them, the *ycf1* and *rps19* are partially duplicated), eight tRNA genes, and all four rRNA genes. Among the 113 genes, 16 contain one intron (10 protein-codes genes, and six rRNA), and three contain two introns (*clpP, ycf3* and *rps12*) (Table 1).

**Table 1:** List of genes identified in the plastome of *Butia eriospatha.*

Group of genes	Name of gene
*Gene expression machinery*	
Ribossomal RNA genes	*rrn4.5* ^*a*^ *; rrn5* ^*a*^ *; rrn16* ^*a*^ *; rrn23* ^*a*^
Transfer RNA genes	*trnA-UGC* ^*ab*^ *; trnC-GCA; trnD-GUC; trnE-UUC; trnF-GAA; trnfM-CAU; trnG-UCC* ^*b*^ *; trnG-GCC; trnH-GUG* ^*a*^ *; trnI-CAU* ^*a*^ *; trnI-GAU* ^*a*^ ^*b*^ *; trnK-UUU* ^*b*^ *; trnL-CAA* ^*a*^ *; trnL-UAA* ^*b*^ *; trnL-UAG; trnM-CAU; trnN-GUU* ^*a*^ *; trnP-UGG; trnQ-UUG; trnR-ACG* ^*a*^ *; trnR-UCU; trnS-GCU; trnS-UGA; trnS-GGA; trnT-UGU; trnT-GGU; trnV-GAC* ^*a*^ *; trnV-UAC* ^*b*^ *; trnW-CCA; trnY-GUA*
Small subunit of Ribossome	*rps2; rps3; rps4; rps7* ^*a*^ *; rps8; rps11; rps12* ^*ab*^ *; rps14; rps15; rps16* ^*b*^ *; rps18; rps19* ^*a*^
Large subunit of Ribossome	*rpl2* ^*ab*^ *; rpl4; rpl16* ^*b*^ *; rpl20; rpl22; rpl23* ^*a*^ *; rpl32; rpl33; rpl36*
DNA-dependent RNA Polymerase	*rpoA; rpoB; rpoC1* ^*b*^ *; rpoC2*
Translational initiation factor	*infA*
Maturase	*matK*
*Genes for photosynthesis*	
Subunits of photosystem I (PSI)	*psaA; psaB; psaC; psaI; psaJ; ycf3* ^*b*^ *; ycf4*
Subunits of photosystem II (PSII)	*psbA; psbB; psbC; psbD; psbE; psbF; psbH; psbI; psbJ; psbK; psbL psbM; psbN; psbT; psbZ*
Subunits of cytochrome b6f	*petA; petB* ^*b*^ *; petD* ^*b*^ *; petG; petL; petN*
Subunits of ATP synthase	*atpA; atpB; atpE; atpF* ^*b*^ *; atpH; atpI*
Subunits of NADH dehydrogenase	*ndhA* ^*b*^ *; ndhB* ^*ab*^ *; ndhC; ndhD; ndhE; ndhF; ndhG; ndhH; ndhI; ndhJ; ndhK*
Large subunit of Rubisco	*rbcL*
*Other functions*	
Envelope membrane protein	*cemA*
Subunit of acetyl-CoA carboxylase	*accD*
C-type cytochrome synthesis	*ccsA*
Subunit of protease Clp	*clpP* ^*b*^
Component of TIC complex	*ycf1*
Unknown function	*ycf2* ^*a*^

a Duplicated genes;

b Genes containing introns

The size of the plastome of *B. eriospatha* was compared with others plastomes from species in the same tribe: Cocoseae (Table 2). They have similar length and structure, varying in the LSC from 83,805 bp to 85,192 bp, the SSC from 17,369 bp to 17,639 bp and the IRs from 26,437 bp to 27,092 bp. This variation can be better visualized in the IRs borders (Figure 2). Species from the subtribes Bactridinae (*Acrocomia* and *Astrocaryum*) and Elaeidinae (*Elaeis*) present the *rps19* gene completely duplicated in the IR region. In contrast, all plastomes sequenced from subtribe Attaleinae (*Butia*, *Cocos*, *Syagrus*) present one complete CDS of *rps19* and one partial copy of *rps19*, thus presenting only one functional copy of the gene (Figure 2). The intergenic spacers (IGS) between *rpl22-rps19* genes and *rps19-psbA* genes, located in the LSC/IR_B_ and IR_A_/LSC junctions, respectively, show variations in size within Cocoseae species (Figure 2). The SSC/IR_A_ junction varies mainly in the length of the *ycf1* gene portion contained within the SSC. The *ndhF* gene is identical in length among all species studied and part of it is located within the IR_B_, overlapping in part the *ycf1* gene (56 bp). The differences in the IR/LSC junctions between Attaleinae and the sister clades Bactridinae + Elaeidinae migth be considered an evolutionary signal and the plastome sequence of *B. eriospatha* may be used in future studies of population genetics and for phylogenetic studies.

**Figure 2:**
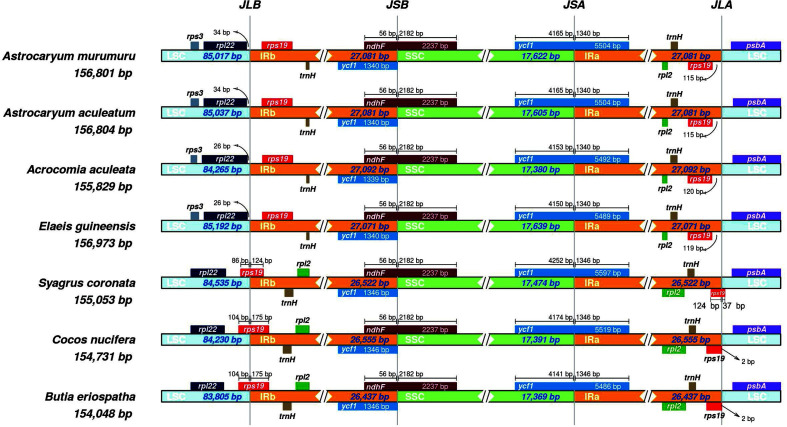
Comparison of plastid genome LSC, IR, and SSC junction positions within the tribe Cocoseae (Arecaceae: Arecoideae). Genes shown below are transcribed counterclockwise and those shown above the lines are transcribed clockwise.

**Table 2:** General features of plastid genomes within the tribe Cocoseae.

Subtribe	Species	Total (bp)	LSC (bp)	IR (bp)	SSC (bp)	Accession number
Attaleinae	*Butia eriospatha*	154,048	83,805	26,437	17,369	MN_329806
	*Syagrus coronata*	155,053	84,535	26,522	17,474	NC_029241
	*Cocos nucifera*	154,731	84,230	26,555	17,391	NC_022417
Elaeidinae	*Elaeis guineensis*	156,973	85,192	27,071	17,639	NC_017602
Bactridinae	*Astrocaryum aculeatum*	156,804	85,037	27,081	17,605	MH_537788
	*Astrocaryum murumuru*	156,801	85,017	27,081	17,622	MH_537787
	*Acrocomia aculeata*	156,500	84,936	27,092	17,380	NC_037084
